# The Impact of Climate Change Awareness on Fertility Intentions in Palestinian Society: Mediating Role of Threat Perception

**DOI:** 10.3390/ijerph22081228

**Published:** 2025-08-06

**Authors:** Maryam W. Fasfous, Mohamed N. Abdel-Fattah, Sarah A. Ibrahim

**Affiliations:** Department of Biostatistics and Demography, Faculty of Graduate Studies for Statistical Research, Cairo University, Cairo 12613, Egypt

**Keywords:** climate change, awareness, fertility intention, threat perception, Palestine

## Abstract

Fertility is considered a significant demographic concern, especially in relation to climate change. This study examines how awareness of climate change, measured by five subscales—climate-friendly behavior, knowledge, personal concern, attitude, and multiplicative action—affects fertility intentions, emphasizing the mediating role of threat perception. Data were collected through an online survey administered to a sample of 817 Palestinian citizens aged 18–49 residing in the West Bank. Structural equation modeling (SEM) was utilized for the data analysis. The results revealed that climate change awareness does not directly affect fertility intentions. However, an indirect effect of climate change awareness on fertility intentions was observed, mediated by threat perception as an intervening variable. Individuals exhibiting increased awareness of climate change and perceptions of future risks demonstrated a greater likelihood of reducing their fertility intentions compared to others. Policymakers in the Palestinian territories should prioritize enhancing public awareness regarding climate change and its associated short- and long-term threats. Therefore, incorporating climate education and associated risks into fertility health programs is essential.

## 1. Introduction

Climate change constitutes a significant global challenge, affecting various aspects of human life, with humans identified as the primary anthropogenic contributor to this issue. The effects encompass demographic changes, mental and physical health, and economic stability [1,2,3,4]. Recent studies have concentrated on population dynamics in the context of climate change, highlighting its impact on demographic variables, including fertility, migration, and mortality [5,6,7]. These variables are significant because of their implications for population size and distribution in the future [8]. Human fertility plays a vital role in population dynamics at both the national and global levels, which is fundamental for long-term strategic planning as well as policy formulation [9,10,11]. Demographic studies have focused predominantly on the impact of climate on migration [12,13] and mortality [14,15,16]. In contrast, a few studies have investigated the connection between fertility and climate issues [17,18,19].

Studies have utilized secondary data to determine the direct and indirect effects of extreme weather conditions on fertility intentions. These studies focused on studying the impact of climate variables, such as rainfall and temperature, on fertility rates over time [20,21]. Furthermore, certain studies have utilized cross-sectional data to investigate the correlation between climate change and individuals’ fertility intentions [22], whereas other studies have utilized cross-sectional data [23]. Research on the influence of climate change on fertility intentions has predominantly concentrated on Western countries, such as the United States [24,25], Canada [26], New Zealand [24], Sweden [17], and Switzerland [27]. Several studies have demonstrated that individuals who express environmental concern are more likely to have altered fertility intentions as a response to climate change. This highlights the significant impact of climate change [26,28,29]. Surveys conducted in Australia and the USA revealed that climate change directly and indirectly impacts fertility intentions [30,31].

While research on fertility intentions and climate change is growing, there remains a paucity of studies within the Middle East, particularly Arab countries. This study examines climate change awareness, its influence on future risk perception, and its direct and indirect effects on fertility intentions. In addition, the study examines a larger population, moving beyond specific demographic types, thereby addressing a significant gap in the context of previous research that has targeted a particular population. Some studies have focused solely on married females [19], whereas other studies have included both males and females [23]. Some studies have focused on individuals exhibiting pronounced climate anxiety [28]. In addition, the study by Fu et al. [32] examined the correlation between climate concerns and reproductive attitudes, thereby elucidating the tensions between environmental concerns and fertility intentions. Helm et al. [25] examined the desire not to have children as an indicator of environmental anxiety, emphasizing how climate change concerns can affect fertility intentions.

The Middle Eastern region has recently experienced climate change, such as droughts, reduced rainfall, and higher temperatures [33,34,35]. This has severely impacted agricultural productivity, water security, and public health [36,37]. Consequently, populations have grown more aware of the risks linked to these changes [18], leading to a notable rise in individual concern and awareness regarding climate change and its effects on the planet and the population [38]. This heightened awareness significantly influences lifestyle and behavioral intentions [39,40] and enables individuals to reconsider their attitudes [41] and make informed decisions to promote engaging in climate-related risk adaptation [42]. Given the increasing concern about environmental issues, psychosocial factors may play a significant role in determining fertility intentions [43], mediating between fertility intentions and awareness of climate change. Limiting the intended number of children is hard unless individuals recognize present or expected climate threats to themselves or their prospective children.

The present study investigates the impact of individuals’ awareness of climate change on their fertility intentions, considering variations in demographic characteristics, eco-anxiety, and mediating variables that may affect fertility intentions. This study investigates an environmental factor as an indicator of fertility intention, contrasting with previous research in the Palestinian context that has predominantly highlighted political, religious, economic, and cultural factors [44,45]. The study introduces a conceptual model combining psychological as well as environmental factors, determined by a person’s view of future threats, particularly within a complex political system. These contexts are defined by the management of essential resources, including land and water, alongside escalating restrictions on people. These conditions lead Palestinians to perceive future threats more intensely than individuals residing in stable environments. The results of the current study are expected to assist policymakers in reducing climate change impacts, adopting adaptive strategies, and promoting sustainable socioeconomic development.

## 2. Conceptual Framework

**Figure 1:**
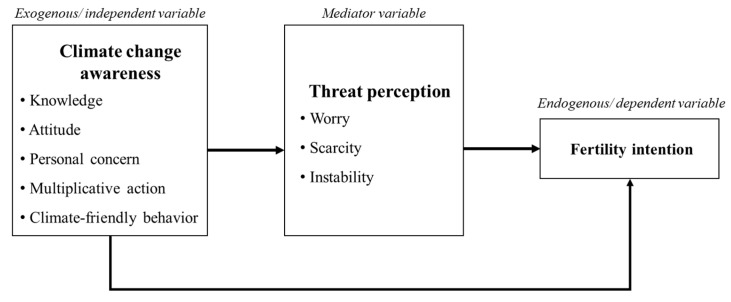
Conceptual model.

Prior research has thoroughly investigated individuals’ awareness of climate change and its effects on various social aspects [46,47,48]. However, research examining the influence of climate change awareness on fertility intentions remains limited. Our analysis involved developing a model based on the current literature. Figure 1 presents the proposed conceptual framework.

Climate change awareness includes cognitive, affective, and conative aspects [49], which influence individuals’ awareness of and attitude towards climate change. The cognitive dimension encompasses an understanding of climate change causes and outcomes [37,50]. The affective aspect encompasses emotional responses elicited by knowledge of climate change [51], while the conative dimension relates to behavioral intentions that emerge from cognitive and affective awareness [39].

A study conducted in Sweden involving 98 participants with varying fertility intentions, age, and level of education investigated the correlation between climate change awareness and fertility intentions. The results demonstrated that although there was significant awareness of climate change, this awareness did not influence fertility intentions [17]. Therefore, our first hypothesis posits that climate change awareness has no direct effect on fertility intentions.

As climate change increases, individuals’ awareness of climatic changes enhances their awareness of associated risks [52,53], encompassing threats to children’s health, safety, and psychological well-being [54]. Multiple studies demonstrate a correlation between increased awareness of climate issues and increased climate anxiety regarding the effects of climate change and future challenges [55,56,57,58]. Individuals’ perceptions of severe climate consequences and associated fears significantly shape future decisions, thereby influencing their fertility intentions [25]. A study in Finland, Estonia, and Sweden found a vital correlation between climate anxiety and fertility intentions, influenced by concerns regarding overpopulation and resource depletion [59]. A survey conducted in the USA involving 607 participants revealed that 586 individuals expressed concerns regarding the quality of life of their future children due to climate change, leading to reduced fertility intentions [60]. Another study involving 334 childless Americans indicated that individuals with significant concerns about climate change demonstrated less favorable pro-reproductive attitudes [25]. Uncertainty regarding the future has a considerable impact on significant life choices, including fertility intentions.

Research consistently indicates that uncertainty and negative perceptions of the future reduce fertility intentions [61,62]. In Northern Europe, increasing apprehensions regarding the future are linked to declining fertility rates [63,64]. Conversely, economic uncertainty reduces fertility intentions in European countries, as concerns about managing climate-induced challenges prompt individuals to opt for a smaller number of children who can have their basic needs met [61,65]. In Arab societies, individual economic concerns have been identified as a factor contributing to declining fertility rates [66].

De Rose et al. [67] found no statistically significant correlation between concerns regarding climate change and the intended number of children, as evidenced by cross-sectional data from the 2011 Eurobarometer survey encompassing 27 European Union countries. Szczuka’s [68] analysis indicated contrasting patterns, as there was a negative correlation between climate change concerns and ideal family size in Slovakia. In contrast, a positive relationship was observed in the Czech Republic. A study involving 200 Canadian university students indicated an inverse relationship between environmental concern and pro-reproductive attitudes. Similar findings were observed in Zambia, where participants’ perceptions of threats from climate-induced livelihood insecurities influenced their decisions to limit childbearing to prioritize essential needs over larger family sizes [69]. The findings indicate that awareness of climate change could increase threat perception, leading to diminished fertility intentions. The relationship is depicted by hypotheses H2 and H3.

Threat perception functions as a mediating variable in examining the impact of climate anxiety on fertility intentions among a sample of 1211 childless Swiss students. Threat perception partially mediated this relationship, indicating that climate anxiety negatively influenced fertility intentions while simultaneously enhancing anti-natalist motivations [27]. The mediating role of threat perception differs across populations and is significantly influenced by the cultural and political factors that shape fertility intentions. The study posits that climate change awareness indirectly diminishes fertility intentions through increased threat perception, as illustrated in H4.

## 3. Materials and Methods

### 3.1. Study Design and Setting

A cross-sectional survey design was used to collect data from 817 Palestinians using an online questionnaire created with Google Forms and disseminated from September to October 2024. The study was conducted in the West Bank, part of the Palestinian-occupied territories. The West Bank is spread across 11 governorates: Jenin, Tubas, Nablus, Salfit, Tulkarm, and Qalqiliya in the north; Jericho and Al-Aghwar, Ramallah and Al-Bireh, and Jerusalem in the middle; and Hebron and Bethlehem in the south. Approximately 76% of the population lives in an area that is very vulnerable to the effects of climate change, which affects around 60% of the land [70].

### 3.2. Study Sample

The study population consisted of Palestinian individuals aged 18–49, defined by the Palestinian Central Bureau of Statistics (PCBS) as the reproductive age group. According to the Population and Housing Census, a total of 1,248,282 Palestinians aged 18 to 49 years were living in the West Bank [71]. A non-probability convenience sample was used to select the study sample due to the political conditions imposed on the West Bank during the study period, which made it most difficult to move between governorates. The sample comprised 817 respondents: 58.6% female, 43.6% holding bachelor’s degrees, and 23.4% holding diplomas. Over 40% of the respondents were aged 26–35. Over 50 percent resided in urban areas (50.1%), while rural areas accounted for 33.9%, and refugee camps comprised 11%. Data on marital status revealed that approximately 70% of the individuals were married, 28% were single, and 2.7% identified as “other.” Approximately 40% of the individuals reported a monthly income between 2500 and 4000 NIS, whereas 32.2% earned below NIS 2500. In addition, 34.8% lived in a contact area with the occupation (Table 1).

### 3.3. Questioner Design

The evaluation of climate change awareness was done utilizing the Climate Change Awareness Scale (CCAS) [49], which consists of 25 items divided into five subscales. The Knowledge subscale comprises four items that assess individuals’ comprehension of the causes and effects of climate change. The Attitude (A) subscale consists of five items that evaluate personal interest in climate change, sense of responsibility to act, commitment to mitigating climate change, and control. The Personal Concern (PC) subscale, evaluated via three items, investigates perceptions regarding the influence of climate change on individual lives, families, and Palestinian societies in the West Bank. The Multiplicative Action (MA) subscale, assessed through four items, measures participation in climate-related discussions with family members and friends. The Climate-Friendly Behavior (CFB) subscale comprises 9 items that evaluate eco-friendly practices performed by individuals. Modifications were implemented to the original scale to improve its cultural and contextual applicability to the sociopolitical and environmental conditions of the West Bank. The evaluation of individuals’ perceptions of threats to current and future environments for children was carried out using the Threat Perception Scale (TPS) [72], comprising 10 items divided into three subscales. The Worry (W) about the future subscale comprises four items that evaluate parental concerns regarding their children’s future. The Scarcity (S) subscale, evaluated via three items, examines perceptions of resource limitations. The Instability (I) subscale, comprising three items, evaluates individuals’ perceptions of the world’s unpredictability. Fertility intention was evaluated by asking the participants, “What is the ideal number of children you wish to have in your lifetime?” [26]. The questionnaire included socioeconomic characteristics such as gender, age, place of residence, and education level, all categorized as categorical variables. The participants were informed about the study’s objectives, assured that participation was voluntary, and guaranteed anonymity, with no collection of personally identifiable information.

### 3.4. Data Analysis

SEM was utilized to assess the conceptual framework; it is a second-generation multivariate analysis technique that facilitates the simultaneous examination of the relationships among latent variables in a study [73]. The partial least squares (PLS) method utilizing Smart-PLS 4 software was used in this study since it is compatible with the exploratory nature of the research. PLS also has no requirement for any assumption regarding distribution [74].

In SEM, the variables are categorized into observable variables (items, indicators), which are directly measured through empirical data collection methods, and unobservable variables (latent variables, constructs), which represent a theoretical concept that cannot be measured directly, but can be measured by multiple indicators.

The conceptual framework was validated through a two-step approach. First, the measurement model was assessed for reliability and validity. Internal consistency was evaluated using Composite Reliability (CR) and Cronbach’s alpha (α), confirming sufficient inter-item correlations within each construct. Convergent validity was established through Average Variance Extracted (AVE > 0.50) and outer loadings (>0.70), demonstrating that items converge on their intended constructs. Discriminant validity was verified via the Fornell–Larcker criterion and Heterotrait–Monotrait (HTMT) ratio (<1.00), ensuring that constructs measure distinct phenomena. Subsequently, the research hypotheses were tested through analysis of the structural model by examining predictive relevance (Q^2^), the coefficient of determination (R^2^), multicollinearity, and effect size (f^2^). Additionally, a multigroup analysis was conducted to compare the conceptual framework across two distinct residence types: areas in contact with occupation zone and areas not in contact with occupation zones.

Jöreskov and Sörbom [75] identify three primary relationships within the SEM framework. The first and second relationships delineate the measurement model, examining the relation between the constructs and their items, while the third relationship outlines the structural model, examining the effect of the exogenous variable on the endogenous variable. The study’s conceptual model incorporates two second-order latent variables: threat perception, serving as the mediating variable, and an exogenous variable represented by awareness of climate change. The mediating variable is assessed through three first-order latent variables: worry, scarcity, and instability. The exogenous variable is measured using five first-order latent variables—climate-friendly behavior, knowledge, personal concern, attitude, and multiplicative action—while the endogenous variable is fertility intention.

The first component of the structural relationships pertains to the measurement model, which evaluates the associations between the observed indicators and the exogenous and mediating constructs at the first-order level. These relationships are represented in Equation (1) and Equation (2), respectively.

(1) $$X=\Lambda_{X\;}\xi +\delta$$

(2) $$Z=\Lambda_{Z}\eta +\varepsilon \;\;\;\;\;\;\;$$

where  X, a vector, represents the observed indicators of the exogenous variable, specifically the dimensions of climate change awareness (climate-friendly behavior, knowledge, personal concern, attitude, and multiplicative action); $\;\Lambda_{X\;}$ denotes a matrix for the regression coefficients; ξ is a random vector for the exogenous variable; and δ is a vector for the error term. Similarly, Z represents the observed indicators of the mediating latent variable, specifically the dimensions of threat perception (worry, scarcity, and instability). $\Lambda_{Z}$ is the regression coefficient matrix, and η the random vector of the mediating latent variable, while ε is a vector representing the error term. Furthermore, it is assumed that ξ, δ, η, and ε are uncorrelated, respectively.

The second part of the measurement model displays the relationships between the second-order latent variable and the first-order constructs and is presented in Equation (3) and Equation (4) for the exogenous and mediating latent variables, respectively.

(3) $$\xi =\Gamma_{X\;}\overset{´}{\xi }+\zeta$$

(4) $$\eta =\Gamma_{Z}\overset{´}{\eta }+\zeta \;\;\;\;\;\;$$

where $\Gamma_{X}$ is the regression coefficient linking the second-order variable (climate change awareness) to its first-order variables (climate-friendly behavior, knowledge, personal concern, attitude, and multiplicative action), and $\Gamma_{Z}$ is the regression coefficient relating the second-order variable (threat perception) to their first-order variable (worry, scarcity, and instability). $\overset{´}{\xi }$ and $\overset{´}{\eta }$ represent the second-order latent variable of the exogenous (climate change awareness) and mediating variable (threat perception), respectively, and ζ is a vector of the error term.

Equations (2) and (4) illustrate the measurement relationships for the first and second parts of the model. The equations capturing the relationships between the observed variables (items) and their respective first-order constructs are derived from Equations (1) and (3) for the exogenous construct, as shown in Equation (5), and from the combination of Equations (2) and (4) for the mediating construct, as presented in Equation (6).

(5) $$X=\Lambda_{X\;}\left(\Gamma_{X\;}\overset{´}{\xi }+\zeta \right)+\delta$$

(6) $$Z=\Lambda_{Z}(\Gamma_{Z}\overset{´}{\eta }+\zeta )+\varepsilon \;\;\;$$

The first relation between the exogenous and mediating variable is defined in Equation (7), Equation (8) defines the second relation between the mediating and endogenous variable is, and the third relation between the endogenous and exogenous variable is defined in Equation (9). These relations specify the causal relation between the study factors in the structural model.

(7) $$\overset{´}{\eta }=\gamma_{1}\overset{´}{\xi }+\zeta_{1}$$

(8) $$Y=\beta \overset{´}{\eta }+\zeta_{2}\;\;\;\;\;\;$$

(9) $$Y=\gamma_{2}\overset{´}{\xi }+\beta \overset{´}{\eta }+\zeta_{2}\;\;\;\;\;\;$$

where $\gamma_{1}$ and $\zeta_{1}$ represent the matrix coefficient of the exogenous variable on the mediating variable and residual term of this relationship, respectively. In Equation (8), Y represents the endogenous variable, and β and $\zeta_{2}$ represent the matrix coefficients of the mediating variable on the endogenous variable and residual term of the relationship, respectively. In Equation (9), $\zeta_{2}$ represents the matrix coefficient.

## 4. Results

### 4.1. Evaluation of the Measurement Model

Composite Reliability and Cronbach’s alpha coefficients were utilized to assess the internal consistency; the values of both coefficients for all constructs exceeded 0.70, thereby meeting the established cutoff value [76]. The outer loading and Average Variance Extracted were used to evaluate the convergent validity. Hair et al. [77] suggested that the model contains items with outer loadings exceeding 0.50. Furthermore, the Average Variance Extracted should be higher than 0.50, according to Fornell et al. [78]. All of the items’ outer loadings were between 0.597 and 0.896, and the values of AVE were between 0.543 and 0.762, since both criteria confirmed that convergent validity is established (see Table 2).

Two criteria were used to evaluate the discriminant validity. First, compare the square root of the Average Variance Extracted indicated on the matrix diagonal with the correlation coefficients between paired constructs indicated on the off-diagonal; according to the Fornell–Larcker criterion [78], the matrix diagonal value is considered to be greater than the values of the off-diagonal in the same column. Second, the ratio of correlation between traits to correlation within traits was found according to Henseler et al. [80]. The ratio values were lower than the conservative threshold of 1.0. Table 3 confirms verifying the discriminant validity.

### 4.2. Evaluation of the Structural Model

After establishing the measurement model, a structural model that estimates the study hypotheses was assessed using multicollinearity, effect size, predictive prevalence, and coefficient of determination.

#### 4.2.1. Multicollinearity

A collinearity test is initially required to evaluate this model. The results in Table 4 confirmed no common method bias, as the Variance Inflation Factors (VIFs) of all constructs are less than 5 [76]. This confirmed that the structural model is not collinear.

#### 4.2.2. Path Coefficient

Hair et al. [76] indicated that the hypothesis was evaluated by a bootstrapping approach using a sub-sample of 5000. The coefficients, *t*-values, and *p*-values for each hypothesis are displayed in Table 5.

**Figure 2:**
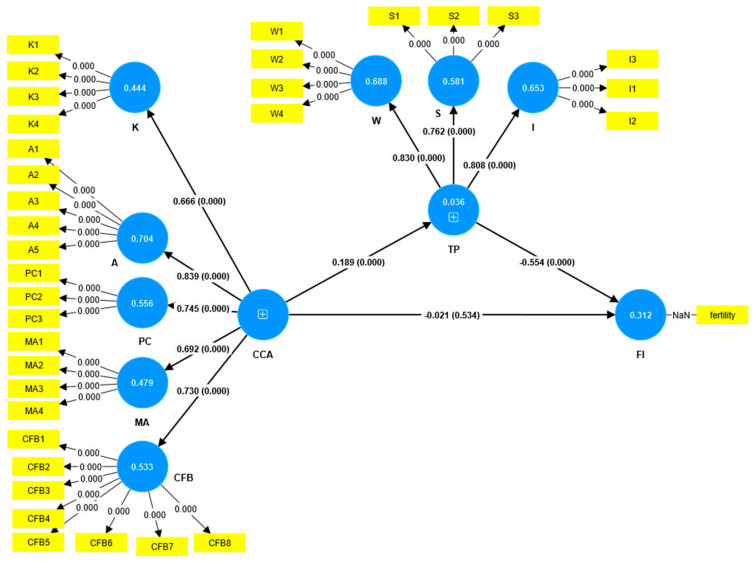
Structural model results. Note: CCA: Climate change awareness; MA: Multiplicative Action; K: Knowledge; A: Attitude; PC: Personal Concern; CFB: Climate-Friendly Behavior; TP: Threat perception; W: Worry; S: Scarcity; I: Instability; FI: Fertility Intention.

The results in Table 5 and Figure 2 show that climate change awareness does not have a significant direct impact on fertility intention, which fails to support the first hypothesis (*p* > 0.05). In contrast, climate change awareness positively influences threat perception, supporting the second hypothesis (*p* < 0.05). Additionally, threat perception significantly negatively affects fertility intention, which supports the third hypothesis: intention (*p* < 0.05). The fourth hypothesis posits that climate change awareness negatively and indirectly affects fertility intention via threat perception; however, this was supported by the findings (*p* < 0.05). This means that threat perception fully mediates the relationship between climate change awareness and fertility intention.

#### 4.2.3. Determination Coefficient, Predictive Relevance, and Effect Size

Assessment of the coefficient of determination ($R^{2}$) and predictive relevance ($Q^{2}$) is an essential aspect of structural model evaluation. $R^{2}\;$ reflects the extent to which the endogenous construct variation can be explained by the entire exogenous construct. A $Q^{2}$ score of zero shows that the exogenous constructions have predictive power [81]. As shown in Table 6, the value of $R^{2}$ indicates that climate change awareness can explain 31.4% of the total variance in fertility intention. Additionally, the $Q^{2}$ value confirms that the structural model exhibits predictive relevance.

After removing the exogenous variables from the study model, their influence on an endogenous variable is evaluated by the effect size ($f^{2}$) [82]. Cohen [83] asserts that a small influence is represented by 0.02, a medium effect by 0.15, and a large by 0.35. The findings in Table 7 indicate that the exogenous variable, climate change awareness, exhibits a small effect size, whereas threat perception demonstrates a significant effect on fertility intention. This suggests that threat perception serves as a significant mediating variable between climate change awareness and fertility intention.

#### 4.2.4. Multigroup Analysis

The multigroup analysis reveals no significant differences in the direct and indirect impact of climate change awareness on fertility intention between contact and non-contact areas (*p*-values = 0.595 and 0.538, respectively). Moreover, awareness of climate change does not significantly influence fertility intentions in either group. In contrast, awareness of climate change has a notable indirect impact on fertility intentions in both groups. This effect is more pronounced in the contact area than in the non-contact area (Table 8).

## 5. Discussion

The present study aimed to examine the direct effect of climate change awareness on fertility intentions, as well as its indirect effect mediated by threat perception in Palestinian society. The study operationalized climate change awareness across five dimensions. The findings indicated an absence of a direct impact of individuals’ understanding of climate change on their fertility intentions. This finding is aligned with the research conducted by Bodin and Björklund [17], who also found no direct effect of climate change awareness on reproductive decisions within Swedish society. This study posits that fertility intentions are closely associated with life choices and social factors in which parenthood is culturally highly regarded. Women with children are frequently perceived more favorably than those without children. They emphasized that environmental responsibility is primarily exhibited through pro-environmental behaviors rather than through reductions in fertility.

This finding arises from the intricate factors influencing reproductive decisions in Palestinian society [44,84], encompassing demographic variables as well as political, cultural, and religious factors [85,86]. Sociopolitical and religious challenges have a greater influence on fertility intentions than environmental concerns, even in light of the growing awareness of climate change and its diverse effects. Decisions regarding family size are mainly linked to the cultural, political, and religious context of Palestinian society, as noted by Pell [85]. Fertility intentions among Palestinians are primarily shaped by sociostructural forces rather than environmental factors, regardless of demographic characteristics.

Childbearing is often described as a form of resistance [87] and an expression of collective identity within the political struggle over Palestinian land [88,89]. Analyses of Palestinian census data from 1997 and 2007 in the West Bank indicated higher fertility rates in regions significantly impacted by occupation, particularly near checkpoints or settlements [45,90]. Religiously, Palestinian Muslim women prioritize adherence to Islamic teachings that discourage limiting family size, focusing instead on birth control methods. They cite Quranic verses to support their opposition to fertility restriction, opting instead for childbearing throughout their reproductive years while using temporary contraceptive measures [85]. Culturally, bearing multiple children, particularly sons, enhances the social status of parents, particularly mothers, who may experience stigma for not producing male heirs. This pressure drives women to persist in childbearing until they have sons, thereby gaining familial respect and marital stability [44,91,92].

The study revealed a positive correlation between climate change awareness and the perception of future risks, while indicating a negative relationship between threat perception and fertility intentions. These findings are consistent with Clayton’s study [93], which suggests that increased knowledge of the negative effects of climate change increases individual anxiety and encourages mitigation actions. Climate change awareness has been associated with different mental health problems in both the short and long term, including panic attacks, feeling anxious, trouble sleeping, and feelings of irritability [94]. In the Palestinian context, where politics are unstable and resources are limited, psychological burdens might increase. They might cause individuals to fear parenthood or stress about their own and their children’s futures, which can affect their fertility intentions and long-term life plans. The mediating role of threat perception in diminishing childbearing desires is further substantiated by research that connects environmental fears to family planning decisions. Individuals with increased climate anxiety demonstrate reduced tendencies to pursue large families or to have children, motivated by concerns about the future welfare of their children [27,59].

This result aligns with research in environmental psychology, which indicates that anxiety about climate-related disasters, including floods, droughts, and extreme heat, may lead individuals to delay parenthood or reduce their desired number of children [95]. Concerns about ecological instability often lead to cautious reproductive planning, as potential parents weigh the ethical and practical implications of raising children. The adverse impact of fertility intentions on reproductive choices can be examined through the lens of economic threats, where economic instability, demonstrated by fluctuating incomes, reduces societal capacity to respond to or mitigate environmental challenges. This is exacerbated by occupational practices that limit access to resources. Economic pressures heighten perceptions of future risks, often negatively influencing fertility intentions due to uncertainty about the ability to provide for future generations [64].

The significance of the mediating model in this study is due to its ability to clarify the relationship between climate change awareness and reproductive behavior via threat perception. The findings indicate that awareness alone does not directly reduce fertility intentions. Instead, its impact is mediated by heightened threat perception, which translates into anxiety about the future and subsequently dampens childbearing decisions. This is consistent with theoretical frameworks in environmental psychology, which suggest that awareness of a particular issue, such as climate change, does not directly influence behavior. It generally navigates cognitive and affective mediators, including anxiety, perceived threats, and moral obligations, before manifesting as actionable behaviors [93,96].

A study involving 360 Palestinians indicated that 26% were “concerned” or “very concerned” about climate change, while 19.7% reported being “terrified” or “very terrified” [97], despite Palestine’s contribution of less than 0.01% to global greenhouse gas emissions [98]. More than 50% of the population is subjected to significant environmental and climatic risks [98], particularly in rural areas, where a considerable number of residents have lost their agricultural livelihoods [99]. This has prompted numerous workers to pursue job opportunities in the Israeli labor market [100]. After the last Gaza War, a significant number of Palestinian workers experienced job losses in this sector [100,101], intensifying concerns regarding their own and their children’s futures in the context of increasing unemployment and limited alternatives in the Palestinian labor market. Consequently, certain individuals have delayed childbearing until the end of the war or reached a state of improved socioeconomic stability.

The study also revealed no significant difference in the direct or indirect effects of climate change awareness on fertility intentions between residents living in direct contact zones affected by occupation and those in non-contact areas. This indicates that awareness of climate change indirectly affects reproductive desires in various residential contexts. This finding is attributed to the widespread human rights violations and systemic insecurity faced by West Bank residents during the Gaza War, which transcends occupation zones. Following October 7, threat perception, including resource scarcity, future anxiety, and instability, increased, as evidenced by elevated path coefficients in areas adjacent to the conflict. Both conflict and non-conflict zones exhibited comparable patterns of threat perception and its negative effect on fertility intentions.

## 6. Conclusions

The findings of the current study indicate a positive impact of climate change awareness on threat perception, suggesting that individuals with increased awareness of climate change are more likely to perceive elevated future risks. The results indicate that increased perception of future threats is associated with decreased fertility intentions; specifically, as individuals’ anxieties regarding climate-related threats rise, their fertility intentions decline. This study identified future threat perception as the primary mediator in the relationship between climate change awareness and fertility intention, indicating that the effect of climate awareness on fertility intention occurs indirectly through increased threat perceptions.

This study was limited by difficulties in acquiring a representative sample of the Palestinian population in the West Bank. It acknowledges potential biases associated with subject self-selection and the utilization of online questionnaires, which may disproportionately represent people who demonstrate heightened interest in climate change and fertility issues, as well as younger, more educated individuals or those with internet access. Additionally, the political conditions in the region hindered the application of probability sampling methods. The study advocates for the establishment of comprehensive policies aimed at enhancing public climate change awareness and its diverse impacts. It emphasizes the significance of fostering pro-environmental behaviors while taking into account social as well as local cultural contexts. The recommendation includes the initiation of a National Climate Education Program, led by the Environmental Quality Authority in collaboration with the Ministries of Education, Higher Education and Scientific Research, and Health. This program would involve extensive community-based workshops, especially among refugee camps, universities, municipalities, and schools. These workshops would provide practical training in waste recycling and urban gardening, connecting these practices to family resilience and future security, thereby reconsidering climate action as a means to achieve well-being and stability.

Furthermore, the incorporation of climate education into reproductive health services is essential. The Ministry of Health and the Palestinian Medical Relief Society (PMRS) should integrate climate awareness into current counseling protocols within primary healthcare clinics. Medical professionals require training to understand the detrimental impact of environmental stressors, including heatwaves, food insecurity, and water scarcity, on child and maternal health. This allows individuals to make mindful decisions regarding reproduction that correspond with their respective abilities and expected environmental challenges, focusing on empowerment instead of population control.

Simultaneously, establishing strategic partnerships with local NGOs is essential. Such collaborations would enable the training of community leaders as “Climate and Health Ambassadors,” who can conduct culturally sensitive discussions on the relationship between environmental conditions, long-term family planning, and reproductive health.

Finally, managing the mental health aspects of climate awareness is crucial. The psychological strain, commonly expressed as climate-associated anxiety, is expected to be heightened in politically unstable environments, including Palestine. Incorporating mental health and psychosocial support (MHPSS) into climate adaptation policies via community-based support groups and training for healthcare professionals enhances overall resilience and promotes a sense of autonomy among individuals confronting ecological disorientation.

In contrast, the study recommended that future research employ a longitudinal design to investigate the long-term effects of climate change on fertility intentions and use both quantitative and qualitative methods to assess these effects. Additionally, to provide a more comprehensive understanding, the study suggested including cultural and political factors that are not fully covered.

## Figures and Tables

**Table 1:** Demographic characteristics sample (N = 817) and population.

	Frequency (N)	Sample %	Population ^a^ %
Gender			
Male	338	41.4	50.98
Female	479	58.6	49.02
Marital status			
Single	228	27.9	
Divorced	16	2.0	
Married	567	69.4	
Widowed	6	0.7	
Age			
18–25	221	27.1	36.37
26–35	358	43.8	32.21
36–49	238	29.1	31.42
Education level			
High school or less	126	15.4	
Diploma	191	23.4	
Bachelor’s	356	43.6	
Postgraduate	144	17.6	
Place of residence			
City	450	55.1	70.80
Village	277	33.9	24.19
Camp	90	11.0	5.01
Monthly average income			
Less than NIS 2500	263	32.2	
NIS 2500–4000	325	39.8	
NIS 4001–6500	126	15.4	
More than NIS 6500	103	12.6	
Residential area			
Contact area with the occupation	284	34.8	
Non-contact area with the occupation	533	65.2	

Note: Percentages that were excluded in the population characteristic were not available in the survey report for the specific age group targeted. ^a^ Population, Housing and Establishments Census 2017 [71].

**Table 2:** Results of internal consistency and convergent validity.

Indicators and Constructs	Outer Loading	CA	CR	AVE
First-order constructs				
Knowledge [K]		0.735	0.834	0.557
K1	0.681			
K2	0.755			
K3	0.798			
K4	0.747			
Attitude [A]		0.832	0.882	0.600
A1	0.699			
A2	0.822			
A3	0.757			
A4	0.774			
A5	0.815			
Personal Concern [PC]		0.727	0.736	0.545
PC1	0.886			
PC2	0.896			
PC3	0.836			
Multiplicative Action [MA]		0.844	0.906	0.762
MA1	0.819			
MA2	0.840			
MA3	0.882			
MA4	0.854			
Climate-Friendly Behavior [CFB]		0.896	0.918	0.586
CFB1	0.748			
CFB2	0.788			
CFB3	0.821			
CFB4	0.666			
CFB5	0.864			
CFB6	0.772			
CFB7	0.845			
CFB8	0.579			
Worry [W]		0.850	0.899	0.690
W1	0.817			
W2	0.869			
W3	0.833			
W4	0.804			
Scarcity [S]		0.745	0.855	0.664
S1	0.747			
S2	0.830			
S3	0.864			
Instability [I]		0.778	0.871	0.693
I1	0.868			
I2	0.863			
I3	0.762			
Second-order constructs				
Climate change awareness [CCA] *		0.794	0.855	0.543
K	0.666			
A	0.839			
PC	0.745			
MA	0.692			
CFB	0.730			
Threat perception [TP] *		0.724	0.842	0.641
W	0.830			
S	0.762			
I	0.808			

Note: CA, CR, and AVE represent Cronbach’s α coefficient, Composite Reliability, and Average Variance Extracted, respectively; * means the values of CA, CR, and AVE were calculated using Excel [79].

**Table 3:** Results of discriminant validity.

	A	CCA	CFB	K	MA	PC	TP	W	S	I
Fornell–Larcker Criterion										
A	**0.775**									
CCA	0.839	**0.737**								
CFB	0.466	0.730	**0.766**							
K	0.556	0.666	0.318	**0.746**						
MA	0.473	0.692	0.315	0.314	**0.849**					
PC	0.585	0.745	0.319	0.467	0.535	**0.873**				
TP	0.118	0.189	0.12	0.131	0.152	0.201	**0.801**			
W	0.144	0.206	0.123	0.117	0.178	0.217	0.830	**0.831**		
S	0.062	0.107	0.065	0.073	0.079	0.133	0.762	0.413	**0.815**	
I	0.061	0.125	0.091	0.120	0.091	0.118	0.808	0.469	0.519	**0.832**
Heterotrait–Monotrait ratio (HTMT)										
A										
CCA	0.937									
CFB	0.531	0.844								
K	0.702	0.809	0.382							
MA	0.552	0.757	0.352	0.390						
PC	0.696	0.814	0.363	0.585	0.623					
TP	0.142	0.212	0.138	0.162	0.175	0.234				
W	0.170	0.228	0.141	0.141	0.210	0.254	0.968			
S	0.083	0.133	0.088	0.112	0.095	0.164	0.963	0.516		
I	0.079	0.144	0.103	0.148	0.104	0.139	0.977	0.564	0.677	

Note: Diagonals in bold represent the square root of each construct AVE. Off-diagonal represents the constraint’s correlation; CCA is a second-order construct of five first-order constructs (K, A, PC, CFB and MA); TP is a second-order construct of three first-order constructs (W, S and I).

**Table 4:** Variance Inflation Factor results.

	VIF
CCA -> FI	1.037
TP -> FI	1.037

**Table 5:** Path coefficients of the research hypotheses.

No.	Path	Coefficient (β)	STDEV	*t*-Value	*p*-Value	Result
1	CCA -> FI	−0.021	0.043	0.622	0.534	Not supported
2	CCA -> PT	0.189	0.043	4.419	0.000 *	Supported
3	TP -> FI	−0.554	0.029	19.091	0.000 *	Supported
4	CCA -> TP -> FI	−0.105	0.025	4.219	0.000 *	Supported

Note: * indicates a significant relation at 1%; STDEV: Standard deviation.

**Table 6:** $Q^{2}$ and $R^{2}$ value.

	$R^{2}$	$Q^{2}(1-\frac{Total\;Sum\;of\;Square}{Sum\;of\;Square\;due\;to\;error})$
Fertility intention	0.314	$0.303(1-\frac{817}{569.699})$

**Table 7:** Results of effect size.

Paths	$\;f^{2}$	Effect Size
CA -> TP	0.041	Small effect
CA -> FI	0.000	No effect
TP -> FI	0.434	Large effect

**Table 8:** Results of multigroup analysis.

		Contact Areas		Non-Contact Areas		Differences	
No.	Paths	Coefficient (β)	*p*-Value	Coefficient (β)	*p*-Value	Coefficient (β)	*p*-Value
1	CCA-> FI	0.006	0.931	−0.034	0.383	0.040	0.595
2	CCA -> TP	0.224	0.002 *	0.166	0.002 *	0.058	0.526
3	TP -> FI	−0.558	0.000 *	−0.554	0.000 *	−0.003	0.938
4	CCA-> TP-> FI	−0.125	0.004 *	−0.092	0.003 *	−0.033	0.538

Note: * indicates a significant relation at 1%.

## Data Availability

The data presented in this study are available upon reasonable request from the corresponding author due to ethical and privacy restrictions and a thesis embargo. The data are part of an ongoing doctoral dissertation and an additional manuscript under preparation; requests for controlled access will be considered after the thesis defense.
